# Nintedanib ameliorates oxidized low-density lipoprotein -induced inflammation and cellular senescence in vascular endothelial cells

**DOI:** 10.1080/21655979.2022.2036913

**Published:** 2022-03-02

**Authors:** Ling Li, Yudan Chen, Chang Shi

**Affiliations:** a Nursing Department, Wuhan Xinzhou District People’s Hospital, Wuhan, China; b Department of Surgery, Wuhan Xinzhou District People’s Hospital, Wuhan, China; c Department of Integrated Traditional and Western Medicine, Wuhan Xinzhou District People’s Hospital, Wuhan, China

**Keywords:** Atherosclerosis, Nintedanib, HUVECs, cell senescence, Arg‐II

## Abstract

Atherosclerosis (AS) is a life-threatening cardiovascular disease and it has been reported that endothelial dysfunction is the initial inducer of AS. Recent reports suggest that inflammation and oxidative stress-induced cell senescence are main inducers of endothelial dysfunction. Nintedanib is an effective inhibitor of multityrosine kinase receptors developed for the treatment of fibrosis, which was recently reported to exert inhibitory effects against inflammation and oxidative stress. The present study plans to study the effect and mechanism of Nintedanib on endothelial dysfunction. We found that in oxidized low-density lipoprotein (ox-LDL)-treated human umbilical vein endothelial cells (HUVECs), the increased production of total cholesterol (TC), free cholesterol (FC), and pro-inflammatory cytokines were observed, reversed by 10 μM and 25 μM Nintedanib. The elevated reactive oxygen species (ROS) and malondialdehyde (MDA) levels, as well as the declined activity of glutathione peroxidase (GSH-Px) in ox-LDL-treated HUVECs, were significantly abolished by 10 μM and 25 μM Nintedanib. Increased proportion of senescence-associated β-galactosidase (SA-β-gal) positive staining cells, activated p53/p21 pathway, and promoted cell fraction in the G0/G1 phase were observed in ox-LDL-treated HUVECs, all of which were dramatically reversed by 10 μM and 25 μM Nintedanib. Lastly, the increased expression level of Arginase-II (Arg-II) in HUVECs by ox-LDL was repressed by Nintedanib. The protective effects of Nintedanib on ox-LDL- induced cellular senescence were pronouncedly blocked by the overexpression of Arg-II. Collectively, our data suggest that Nintedanib mitigates ox-LDL-induced inflammation and cellular senescence in vascular endothelial cells by downregulating Arg‐II.

## Graphical abstract

**Figure:**
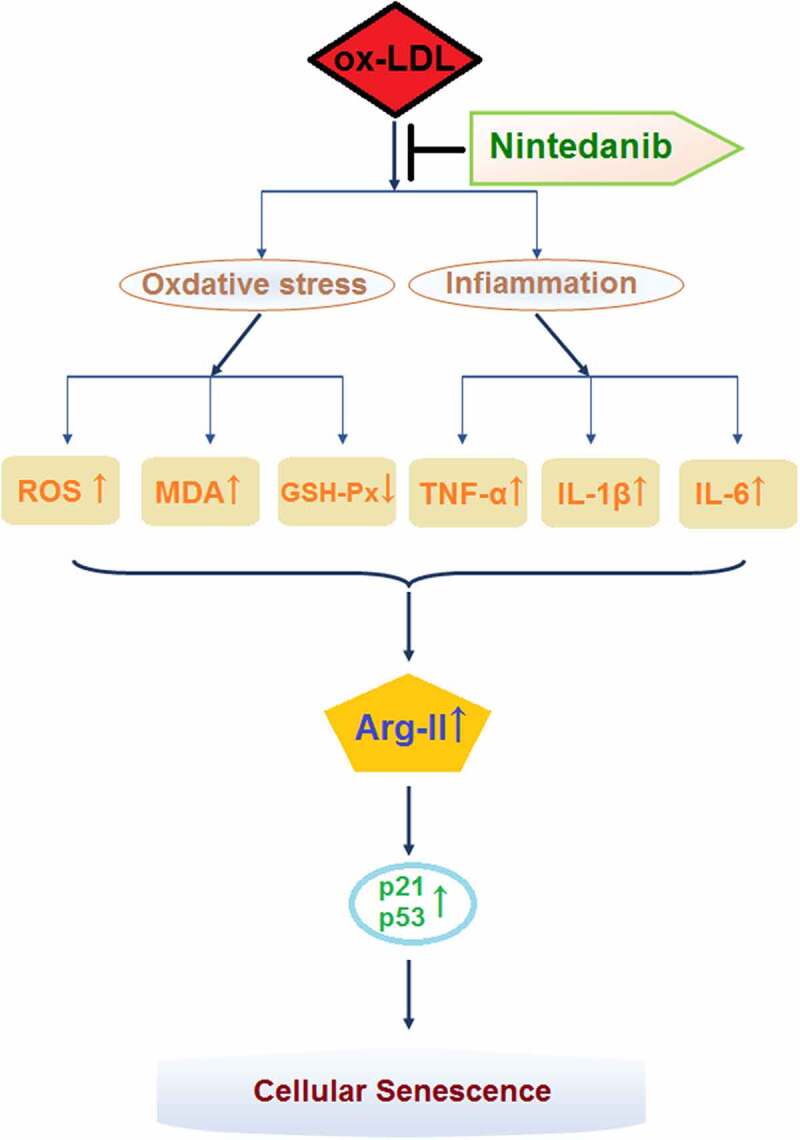

## Introduction

Endothelial dysfunction is considered the initial inducer of atherosclerosis (AS) and has been proven to be observed in the subclinical stage of AS [1], which is defined as various non-adaptive changes in endothelial cell function that impact hemostasis, local vascular tension, REDOX balance, and acute or chronic inflammatory responses [2]. Endothelial dysfunction can be detected in the inflammatory site and AS injury site, and the permeability of dysfunctional endothelial cells to low-density lipoproteins (LDLs) in the blood is significantly increased. The absorbed LDL is transformed into oxidized LDL (ox-LDL), a damage-related molecular pattern (DAMP) which further induces injuries on endothelial cells by triggering inflammatory processes [3]. Damaged endothelial cells release excessive cytokines, chemokines, and adhesion molecules. These recruit monocytes in circulating blood into the AS site and induce the transformation of monocytes into pro-inflammatory macrophages (M1 macrophages). Pro-inflammatory factors, such as interleukin-1β (IL-1β), interleukin-6 (IL-6), and tumor necrosis factor-α (TNF-α), are then secreted by M1 macrophages and foam cells, aggravating the progression of AS [4]. It is recently reported that cell senescence, which can be triggered by multiple diseases such as hypertension, hyperlipidemia, and diabetes, is responsible for the development of endothelial dysfunction [5]. Pro-inflammatory cytokines including IL-1β and TNF-α are well-known important risk factors for cell senescence in various diseases [6,7]. The length of telomeres can be regulated by multiple elements, including Cytokinins, inflammatory factors, angiotensin II [8], oxidants and antioxidants [9], and nitric oxides (NO) [10], achieved by changing the concentration of oxidative stress products [11]. 8-hydroxydeoxyguanosine (8-oxodG) is a sensitive biomarker produced after oxidative stress damage to DNA and the degree of oxidative stress can be reflected by its concentration. Under oxidative stress, the level of 8-oxodG is relatively high at the site of the telomere. Additionally, as the repairability of telomeres against oxidative stress is weaker than other parts of chromosomes, more severe damage is induced by extracellular reactive oxygen species (ROS) on telomere length. Regulating cell senescence in endothelial cells by alleviating oxidative stress might be a promising method to treat AS. Arginase-II (Arg-II), mainly expressed in the mitochondria of cells, could increase mitochondrial ROS production, triggering inflammatory response [12]. Xiong *et al*. found that Arg-II induces vascular smooth muscle cell senescence in both *in vivo* and *in vitro* AS models [13]. These findings demonstrated the association of Arg-II with cellular senescence in AS, indicating that Arg-II might be a potential target for the treatment of AS.

Nintedanib is an effective inhibitor of multityrosine kinase receptors such as platelet-derived growth factor receptor (PGGFR), fibroblast growth factor receptor (FGFR), and vascular endothelial growth factor receptor (VEGFR). It represses the progression of fibrosis by regulating multiple signaling pathways [14]. The anti-fibrosis efficacy of Nintedanib has been proven both in the laboratory [15] and clinical trials [16]. Recently, significant inhibitory effects of Nintedanib against oxidative stress [17] and inflammation [15] have been reported. The present study aims to investigate the effects of Nintedanib on ox-LDL-induced endothelial dysfunction to provide the fundamental basis for the potential application of Nintedanib to treat AS.

## Materials and methods

### Cell culture, treatment, and transduction

Human umbilical vein endothelial cells (HUVECs) were obtained from Shanghai Honsun Biological Technology Co., Ltd (Shanghai, China), and incubated in DMEM medium containing 10% FBS and penicillin-streptomycin. The incubation system of cell culture was 5% CO_2_ and 37°C. To construct Arg-II overexpressed HUVECs, cells were transfected with the pcDNA3.1-Arg-II plasmid together with lipofectamine 3000 (Thermo Fisher Scientific, Massachusetts, USA), followed by verification using the Western blotting method after 24-hour incubation.

### Cell counting kit-8 (CCK-8) assay

HUVECs were implanted in the 24-well plate, followed by being treated with different concentrations of Nintedanib (1, 5, 10, 25, 50, and 100 μM) for 24 hours. Subsequently, CCK-8 solution was added to each well and incubated for 4 hours, followed by determining the optical density (OD) at 450 nm using the microplate reader (Bio-Chain, Shanghai, China) [18].

### Total cholesterol and free cholesterol

The concentration of total cholesterol and free cholesterol released by HUVECs was determined using the commercial kit, Cholesterol/Cholesteryl Ester Quantitation Kit (Biovision, California, USA), according to the instruction described by the manufacturer [19].

### Real-time PCR

The Trizol reagent (Absin, Shanghai, China) was utilized to isolate RNA from treated HUVECs, followed by converting RNA to cDNA using the Prime Script RT reagent kit (Takara Bio, Shiga, Japan). The SYBR Green system (Applied Biosystems, California, USA) and an ABI 7500 (Applied Biosystems, California, USA) were used to perform the real-time PCR. The normalization of the housekeeping gene β-actin was conducted to calculate the relative expression level of targeted genes, followed by determining the fold changes using the 2^−ΔΔCt^ method.

### Enzyme-linked immunosorbent assay (ELISA)

ELISA assay was used to detect the release of TNF-α, IL-8, and IL-6 (Elabscience, Wuhan, China). Briefly, the collected supernatants and standards were centrifuged at 300 × g for 5 minutes and further implanted in a 96-well plate for 1 hour incubation at 37°C, followed by removing samples and washes using PBS buffer. After adding the conjugate reagents to be incubated for 30 minutes, TMB solution was added to the wells. Then, the reaction was stopped by introducing the stop solution. The OD values at 450 nm were detected using the microplate reader (Bio-Chain, Shanghai, China).

### Dichloro-dihydro-fluorescein diacetate (DCFH-DA) assay

In brief, HUVECs were added with DCFH-DA reagent (ADANTI, Wuhan, China) to be incubated for 10 minutes at 37°C and the serum-free DMEM medium was used to wash the remaining reagent [20]. The release of ROS was quantified with the ImageJ software after visualization utilizing the inverted fluorescence microscope (KEYENCE, Shanghai, China).

### Malondialdehyde (MDA) measurements

A commercial kit (Solarbio, Beijing, China) was used to determine the production of MDA by HUVECs. In brief, cells were centrifugated at 8000 g for 10 minutes and then added with the extracting reagent. After adding the MDA detection reagent and fully mixing for 1 hour at 100°C, samples were centrifugated at 10000 g for 15 minutes. Then the supernatant was obtained and implanted into the 96-well plate, followed by measuring the absorbance at 450, 532, and 600 nm using the microplate reader (Bio-Chain, Shanghai, China).

### Glutathione peroxidase (GSH-Px) measurements

A commercial kit (Solarbio, Beijing, China) was used to determine the activity of GSH-Px in HUVECs. In brief, cells were centrifugated at 8000 × g for 15 minutes and then added with the extracting reagent. After adding the corresponding reagents and fully mixing, the absorbance at 412 nm was detected using the microplate reader (Bio-Chain, Shanghai, China).

### Senescence-associated β-galactosidase (SA-β-gal) staining

The cell senescence in HUVECs was evaluated with the SA-β-gal staining assay. In brief, HUVECs were fixed with 4% paraformaldehyde and washed 15 minutes later, followed by adding SA-β-gal staining solution to be incubated overnight at 37°C. Under a light microscope (KEYENCE, Shanghai, China), stained cells were visualized.

### Cell cycle measurement with a flow cytometer

HUVECs were harvested and fixed using 70% cold ethanol at −20°C for 8 hours, followed by being treated with RNase A. After being stained with 10 µg/mL propidium iodide for half an hour, HUVECs were loaded to a flow cytometer (BD, California, USA) for the analysis of the cell cycle [21].

### Western blot analysis

Proteins were extracted from cells and then quantified with a BCA kit (Absin, Shanghai, China), followed by being loaded onto a 12% SDS PAGE. After separating for 1 hour, proteins were transferred onto the PVDF membrane, and further incubated with 5% BSA. Then, the primary antibody against Arg-II (1:2000, LifeSpan, Maryland, USA), p21 (1:1000, LifeSpan, Maryland, USA), p53 (1:1000, LifeSpan, Maryland, USA), and β-actin (1:1000, LifeSpan, Maryland, USA) were added into the membrane, followed by adding the secondary antibody (1:2000, LifeSpan, Maryland, USA) for 90 minutes. After exposure to ECL solution, bands were quantitated using the Image J software.

### Statistical analysis

Data achieved in the present study were presented as mean± standard deviation (S.D.) and analyzed using the software GraphPad. The analysis of variance (ANOVA) method was used to analyze the differences among groups, followed by the Tukey’s test. Two independent data sets were analyzed using Student’s t-test. P < 0.05 was considered a significant difference in the present study.

## Results

In this study, we explored the pharmacological function of Nintedanib in ox-LDL-challenged HUVECs. Firstly, we found that Nintedanib inhibited ox-LDL-induced lipid accumulation in HUVECs. Secondly, Nintedanib ameliorated ox-LDL-induced oxidative stress and inflammatory response by inhibiting the production of ROS, MDA, and the expression of pro-inflammatory cytokines. Nintedanib attenuated ox-LDL- induced cellular senescence and cell cycle arrest in the G0/G1 phase. Importantly, we found that the protective effects of Nintedanib on ox-LDL-stimulated HUVECs were mediated by its inhibition of Arg-II.

### Cytotoxicity of Nintedanib in HUVECs

**Figure 1.**
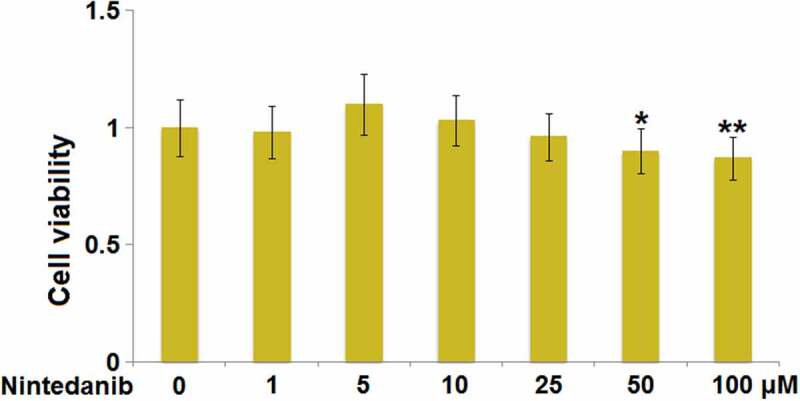
Cytotoxicity of Nintedanib in endothelial cells. Cells were treated with Nintedanib at concentrations of 1, 5, 10, 25, 50, 100 μM. Cell viability was determined with the CCK-8 assay (*, **, P < 0.05, 0.01 vs. Vehicle group).

The cell viability was detected after HUVECs were added with Nintedanib (1, 5, 10, 25, 50, and 100 μM) to determine the optimized incubation concentrations. When the concentration of Nintedanib was greater than 50 μM, a significantly declined viability of HUVECs was observed (Figure 1). In the subsequent experiments, 10 μM and 25 μM Nintedanib were used for the investigations.

### Nintedanib prevented ox-LDL-induced lipid accumulation in HUVECs

**Figure 2.**
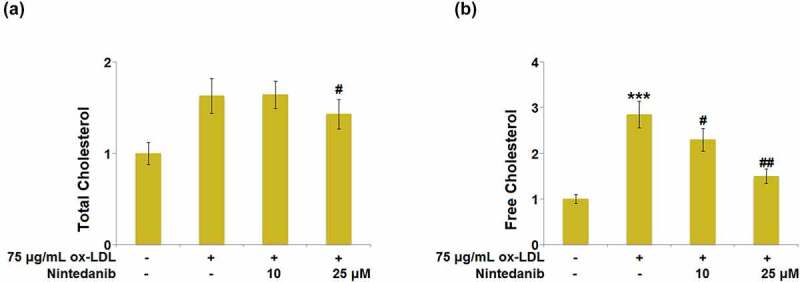
Nintedanib prevents ox-LDL-induced lipid accumulation in HUVECs. Cells were treated with 75 μg/mL ox-LDL with or without 10 μM and 25 μM of Nintedanib for 24 hours. (a). The levels of Total Cholesterol (TC); (b) Free Cholesterol (FC) (***, P < 0.001 vs. Vehicle group; #, ##, P < 0.05, 0.01 vs. ox-LDL group).

Lipid accumulation is a significant output of ox-LDL stimulation in endothelial cells. The level of total cholesterol (TC) and free cholesterol (FC) was measured after cells were treated with 75 μg/mL ox-LDL with or without 10 μM and 25 μM Nintedanib for 24 hours. We found that the release of TC (Figure 2(a)) and FC (Figure 2(b)) was dramatically increased in ox-LDL-treated HUVECs, but repressed by 10 μM and 25 μM Nintedanib.

### Nintedanib suppressed ox-LDL-induced expression of pro-inflammatory cytokines in HUVECs

**Figure 3.**
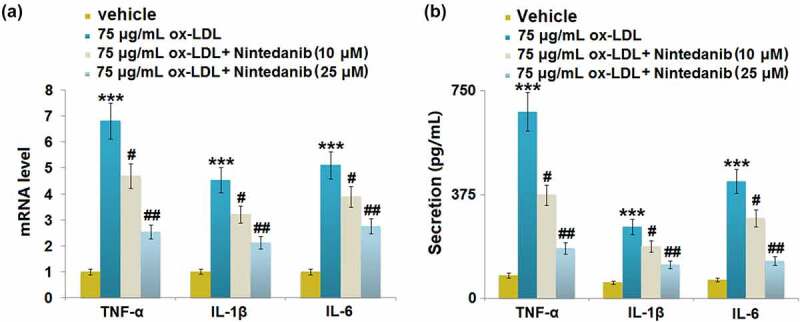
Nintedanib reduces ox-LDL LDL-induced inflammation in HUVECs. (a) mRNA levels of TNF-α, IL-1β, IL-6; (b) Secretion levels of TNF-α, IL-1β, IL-6 (***, P < 0.001 vs. Vehicle group; #, ##, P < 0.05, 0.01 vs. ox-LDL group).

The release of inflammatory factors was measured after cells were treated with 75 μg/mL ox-LDL with or without 10 μM and 25 μM Nintedanib for 24 hours. Upregulated TNF-α, IL-8, and IL-6 were observed in ox-LDL-challenged HUVECs, which were greatly downregulated by 10 μM and 25 μM Nintedanib (Figure 3(a)). The secretion of TNF-α (Figure 3(b)) was elevated from 83.42 pg/mL to 374.31 pg/mL by ox-LDL, then greatly repressed to 253.25 and 161.46 pg/mL by 10 μM and 25 μM Nintedanib, respectively. The production of IL-1β (Figure 3(b)) in the control, ox-LDL, 10 μM, and 25 μM Nintedanib groups was 56.28, 258.35, 187.96, and 121.63 pg/mL, respectively. Additionally, the release of IL-6 was promoted from 66.45 to 423.87 pg/mL by ox-LDL, which was then declined to 289.61 and 135.89 pg/mL by 10 μM and 25 μM Nintedanib, respectively. These results suggest that the inflammation in ox-LDL-treated HUVECs was alleviated by Nintedanib.

### Nintedanib attenuated ox-LDL-induced oxidative stress in HUVECs

**Figure 4.**
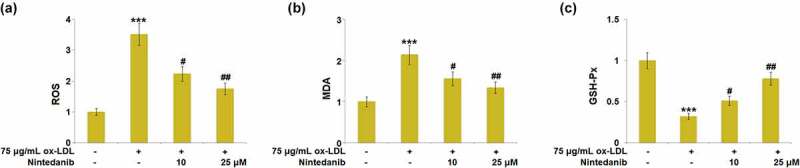
Nintedanib reduces ox-LDL-induced oxidative stress in HUVECs. (a) ROS levels; (b) MDA levels; (c) GSH-Px levels (***, P < 0.001 vs. Vehicle group; #, ##, P < 0.05, 0.01 vs. ox-LDL group).

Oxidative stress is an important inducer for the development of cell senescence in endothelial cells. Subsequently, the levels of oxidative stress biomarkers were measured. Increased release of ROS was observed in ox-LDL-stimulated cells, which was greatly suppressed by 10 μM and 25 μM Nintedanib (Figure 4(a)). The production of MDA was dramatically promoted by ox-LDL, and then greatly declined in 10 μM and 25 μM Nintedanib-treated HUVECs (Figure 4(b)). Inversely, the declined activity of GSH-Px was observed in ox-LDL-stimulated cells but greatly elevated by 10 μM and 25 μM Nintedanib (Figure 4(c)). These results suggest that oxidative stress in HUVECs triggered by ox-LDL was ameliorated by Nintedanib.

### Nintedanib reduced ox-LDL-induced cellular senescence in HUVECs

**Figure 5.**
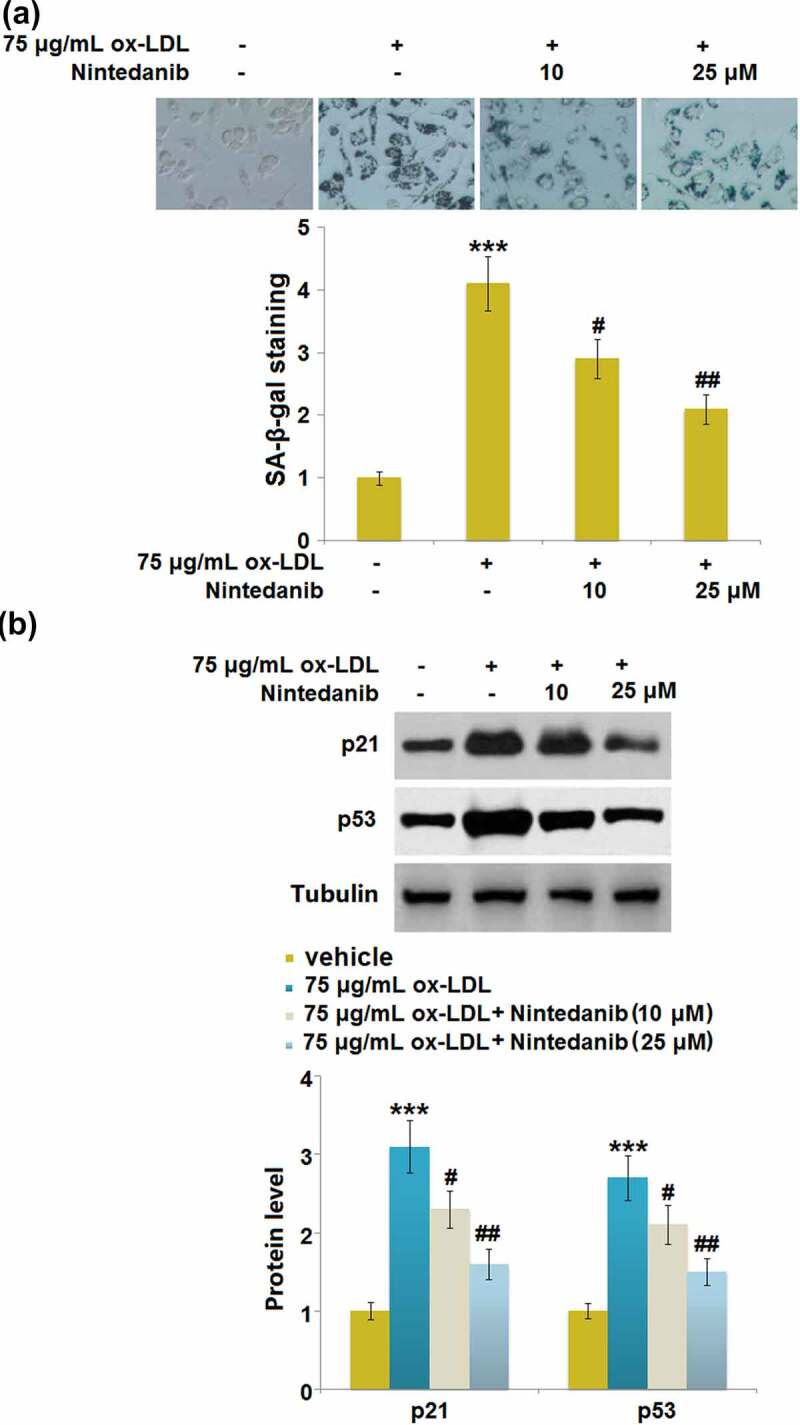
Nintedanib reduces ox-LDL- induced cellular senescence in HUVECs. Cells were treated with 75 μg/mL ox-LDL with or without 10 μM and 25 μM of Nintedanib for 7 days. (a). SA-β-gal staining;(b). Immunoblotting analysis of cellular senescence marker p21 andp53 (***, P < 0.001 vs. Vehicle group; #, ##, P < 0.05, 0.01 vs. ox-LDL group).

Cell senescence is reported to be an inducer for endothelial dysfunction ^5^. We found that the SA-β-gal positive staining in ox-LDL-treated HUVECs was significantly increased but largely declined in 10 μM and 25 μM Nintedanib-treated HUVECs (Figure 5(a)). Additionally, upregulated p21 and p53 were observed in ox-LDL-treated HUVECs, which were greatly reversed by 10 μM and 25 μM Nintedanib (Figure 5(b)). Severe cell senescence in ox-LDL-stimulated cells was dramatically reduced by Nintedanib.

### Nintedanib inhibited ox-LDL-induced cell cycle arrest in G0/G1 phase in HUVECs

**Figure 6.**
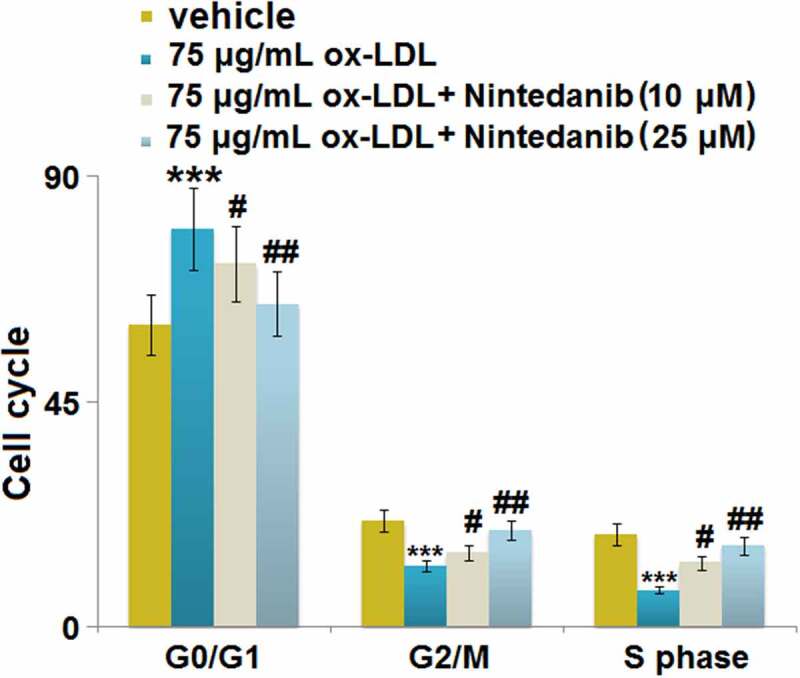
Nintedanib inhibits ox-LDL-induced cell cycle arrest in G0/G1 phase in HUVECs. Cell fraction in the G0/G1phase, G2/M phase, and S phase was calculated (***, P < 0.001 vs. Vehicle group; #, ##, P < 0.05, 0.01 vs. ox-LDL group).

We further explored the impact of Nintedanib on the cell cycle in HUVECs, the inhibition of which is regularly observed under the state of cell senescence [22]. We found that the proportion of G0/G1 phase cells (Figure 6) was significantly elevated from 60.31% to 79.45% by ox-LDL but greatly repressed to 72.41% and 64.52% by 10 μM and 25 μM Nintedanib, respectively. The cell fraction in the G2/M phase (Figure 6) in the control, ox-LDL, 10 μM, and 25 μM Nintedanib groups was 21.18%, 12.21%, 15.82%, and 19.34%, respectively. Additionally, the cell fraction in the S phase (Figure 6) was declined from 18.49% to 7.33% by ox-LDL but greatly elevated to 12.76% and 16.17% by 10 μM and 25 μM Nintedanib, respectively. These data imply that the cell cycle arrest in HUVECs induced by ox-LDL was greatly reversed by Nintedanib.

### Nintedanib inhibited the expression of Arg-II elevated by ox-LDL stimulation

**Figure 7.**
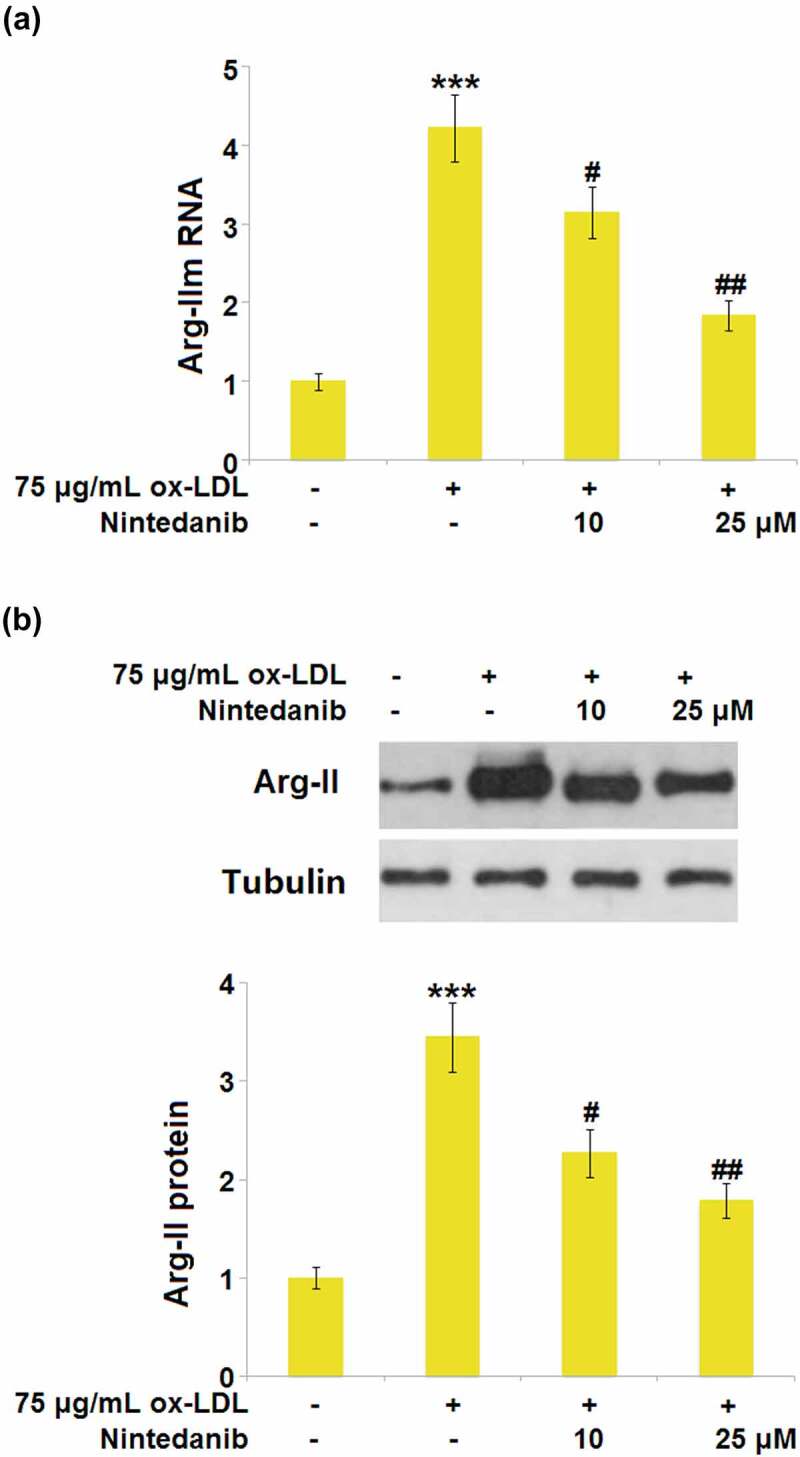
Nintedanib inhibits the expression of Arg-II elevated by ox-LDL treatment in HUVECs. Cells were treated with 75 μg/mL ox-LDL with or without 10 μM and 25 μM of Nintedanib for 24 hours. (a). mRNA of Arg-II; (b). protein level of Arg-II (***, P < 0.001 vs. Vehicle group; #, ##, P < 0.05, 0.01 vs. ox-LDL group).

Arg-II is reported to be an important inducer for the development of cell senescence [13]. We found that a dramatically elevated expression level of Arg-II (Figure 7(a,b)) was observed in the ox-LDL group, which was greatly downregulated by 10 μM and 25 μM Nintedanib, implying the effects of Nintedanib might be associated with the inhibition of Arg-II production.

### Overexpression of Arg-II blocked the protective effects of Nintedanib on ox-LDL- induced cellular senescence

**Figure 8.**
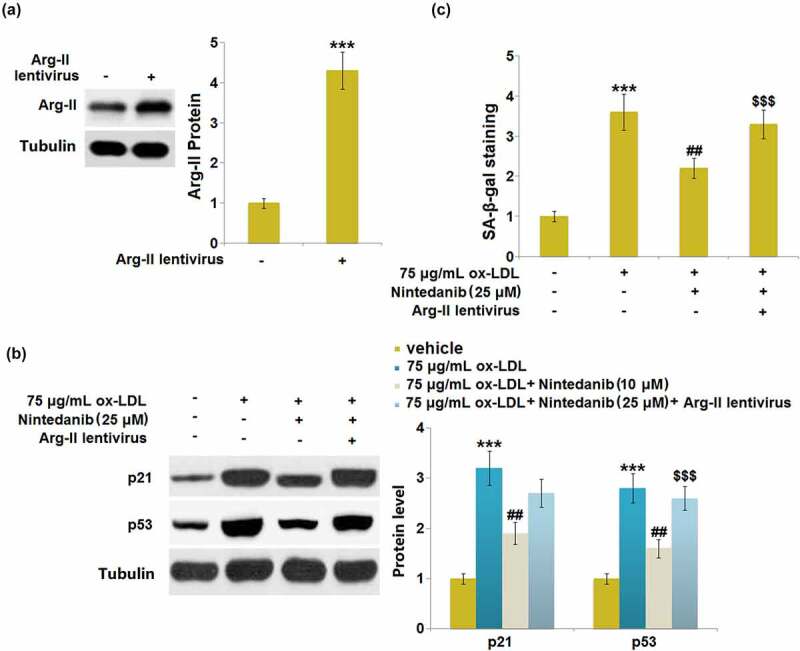
Overexpression of Arg-II blocked the protective effects of Nintedanib on ox-LDL induced and cellular senescence. Cells were infected with Arg-II lentivirus for 48 hours and then treated with 75 μg/mL ox-LDL with or without 25 μM of Nintedanib for 24 hours. (a). Western blot analysis revealed successful overexpression of Arg-II; (b). Immunoblotting analysis of cellular senescence markerp21 andp53;(c). SA-β-gal staining (***, P < 0.001 vs. Vehicle group; ##, P < 0.01 vs. ox-LDL group; $$$, P < .001 vs. ox-LDL+Nintedanib group).

To verify the involvement of Arg-II in the regulation of Nintedanib against cell senescence, Arg-II-overexpressed HUVECs were established. Cells were infected with Arg-II lentivirus for 48 hours and subsequently stimulated with 75 μg/mL ox-LDL with or without 25 μM Nintedanib for 24 hours. Firstly, significantly upregulated Arg-II was observed in Arg-II lentivirus-transfected HUVECs (Figure 8(a)). The elevated expression levels of p21 and p53 in ox-LDL-stimulated cells were greatly repressed by 25 μM Nintedanib, which was abolished by the overexpression of Arg-II (Figure 8(b)). Additionally, the increased proportion of SA-β-gal positive staining in ox-LDL-treated HUVECs was dramatically reduced by 25 μM Nintedanib, which was abolished by overexpressing Arg-II (Figure 8(c)). These data suggest that the effects of Nintedanib on cell senescence were regulated by Arg-II.

## Discussion

Mitochondrial dysfunction caused by free radical oxidative damage is an important mechanism of cellular senescence. Mitochondria are the main sites of free radical generation and are also vulnerable to ROS radical attacks and DNA oxidative damage [23]. Under a normal physiological state, the balance between the production and elimination of ROS is maintained in endothelial cells. However, when the ability to eliminate ROS declines, cell senescence is induced, accompanied by declined respiratory chain effects and reduced production of ATP [24]. Damage and breakage on DNA are then induced by the excessively accumulated ROS, along with the promoted collagen cross-linking and changes in the physical and chemical properties of connective tissue. In the process of aging, the production of ROS is increased, resulting in significant damages to mitochondrial DNA and subsequent accelerated aging. The long-term accumulation of such effects is considered a basis for the occurrence of cellular aging [25,26]. MDA is the terminal product of lipid peroxidation induced by the action of oxygen free radicals on polyunsaturated fatty acids on biofilms. The level of MDA reflects the severity of the free radical attack and can be used as an indicator to evaluate the degree of oxidative stress [27–29]. GSH-Px, a negative biomarker of oxidative stress, can be inactivated in the progression of cell senescence [30]. In HUVECs, we found the activation of oxidative stress and inflammation were dramatically stimulated by ox-LDL, which was consistent with a previous report [31]. After the treatment with Nintedanib, oxidative stress and the production of inflammatory factors were greatly alleviated, suggesting a promising anti-oxidative property of Nintedanib in endothelial cells.

Cell senescence refers to irreversible cell cycle stagnation caused by activation of a series of intracellular signaling pathways under the stimulation of a variety of internal and external factors, which are mainly characterized by chromatin changes, changes in cell metabolism and morphology, and enhanced activity of β-galactosidase [32,33]. The p53/p21 signaling pathway is one of the main mechanisms regulating cell senescence, which is involved in the progression of cell senescence stimulated by multiple factors [34]. P53 is an important tumor suppressor gene that activates the promoters of its target genes, initiating gene transcription, and participating in important processes such as tumor proliferation, apoptosis, and DNA damage. The regulatory effect of p53 on cell senescence is mainly mediated by its target gene p21 [35], which is a member of the Cip/Kip family and induces cell cycle arrest by inhibiting the activity of CDK family proteins [36]. We found that the cell senescence was significantly induced in HUVECs by the stimulation of ox-LDL, accompanied by the activation of the p53/p21 pathway and cell cycle arrest, which is consistent with the observation reported by Zhang in 2018 [37]. After the treatment with Nintedanib, cell senescence was dramatically alleviated, indicating that Nintedanib might ameliorate endothelial dysfunction by reversing the progression of cell senescence.

Arg‐II is an enzyme that catalyzes the hydrolysis of L-arginine to produce ornithine and urea and is reported to be located in the mitochondria [38]. Recently, it has been reported that Arg‐II is involved in the development of cell senescence. Xiong claimed that the cell senescence in vascular smooth muscle cells could be triggered by Arg‐II by regulating the p66Shc and p53 pathways [13]. Zhu et al. reported that the inflammation and senescence in endothelial cells could be facilitated by the stimulation of Arg‐II [39]. A recent study showed that stimulation with IL-1β significantly increased the expression of Arg‐II, indicating that the production of pro-inflammatory cytokines during the inflammatory response could trigger the expression of Arg‐II [40]. Furthermore, silencing of Arg‐II attenuated inflammatory response and oxidative stress, which play a vital role in cellular senescence. We found that Arg‐II in HUVECs was significantly upregulated by ox-LDL, accompanied by the processing of cell senescence. Following the introduction of Nintedanib, Arg‐II was greatly downregulated, implying that Arg‐II might be a mediator for the function of Nintedanib. Verification experiments revealed that overexpression of Arg-II blocked the protective effects of Nintedanib on ox-LDL- induced cellular senescence. The main limitation of this study is that all the findings are based on *in vitro* models. Cellular senescence in AS is a complicated process. Animal experiments are important ways to explore the pathogenesis and treatment of AS. In future work, the mechanism underlying the regulatory effect of Nintedanib on Arg-II will be further investigated in both *in vitro* and *in vivo* models to better comprehend the function of Nintedanib in endothelial senescence and dysfunction.

## Conclusion

Collectively, the present study suggested that Nintedanib ameliorated ox-LDL-triggered inflammation and cellular senescence in vascular endothelial cells by downregulating Arg‐II. These findings suggest that Nintedanib might be a promising agent used for the treatment of AS.

## Data Availability

The data and materials of this study are available upon reasonable request from the corresponding authors.
